# An Enhanced Red Bioluminescent Indicator for Responsive Detection of Physiological Calcium Dynamics in Cells and Mice

**DOI:** 10.1021/acssensors.5c01093

**Published:** 2025-07-29

**Authors:** Xiaodong Tian, Yiyu Zhang, Haoyang Du, Wenyuan Huang, Laurie Anne Bizimana, Nozomi Nishimura, Hui-wang Ai

**Affiliations:** † Department of Molecular Physiology and Biological Physics, 12349 University of Virginia School of Medicine, Charlottesville, Virginia 22908, United States; ‡ Center for Membrane and Cell Physiology, 12349 University of Virginia School of Medicine, Charlottesville, Virginia 22908, United States; § Meinig School of Biomedical Engineering, 5922 Cornell University, Ithaca, New York 14853, United States; ∥ The UVA Comprehensive Cancer Center, 2358 University of Virginia Charlottesville, Virginia 22908, United States

**Keywords:** bioluminescent sensor, calcium imaging, bioluminescence imaging, red-shifted emission, protein engineering

## Abstract

Calcium (Ca^2+^) is a crucial metal ion and signaling messenger. While bioluminescent indicators for Ca^2+^ have emerged as powerful imaging tools, their performance has been suboptimal. In this study, we developed an enhanced bioluminescent red indicator for Ca^2+^ (eBRIC) by using a physiological Ca^2+^ concentration range during library screening. Compared with its predecessors, this new sensor demonstrates substantially improved Ca^2+^ responsiveness in protein-based assays, cultured cell lines, and primary neurons. We further demonstrated the utility of eBRIC for the in vivo recording of Ca^2+^ dynamics in the brains of live mice, using both a microscope setup and a luminescent imaging dark box. Notably, by combining eBRIC with our recently developed water-soluble luciferin, we achieved minimally invasive, video-rate imaging of Ca^2+^ activity in a defined brain region of awake mice. In a footshock-induced basolateral amygdala activation paradigm, eBRIC elicited approximately double the response compared to the previous BRIC indicator. The improved responsiveness offered by eBRIC underscores its potential as a powerful tool for investigating Ca^2+^ dynamics in living systems.

## Graphical abstract

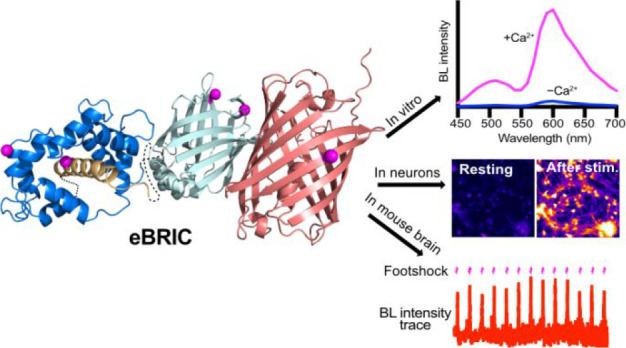

Ca^2+^ is among the most widely recognized secondary messengers, intricately linked to numerous biological processes.^1–3^ Over the years, fluorescent calcium indicators have been developed into powerful research tools, enabling researchers to monitor Ca^2+^ dynamics with high spatiotemporal resolution at both the cellular and subcellular levels.^1,2,4^ Since Ca^2+^ influx serves as a proxy for neuron activation, these Ca^2+^ indicators have been extensively applied to in vivo animal imaging, becoming indispensable for studying brain functions.^5^ However, their use is limited by inherent challenges associated with the requirement for excitation photons. These include phototoxicity to biological samples, interference with photon-sensitive biological processes, and incompatibility with optogenetic tools.^6,7^ Additionally, observing neuronal activity in brain regions often necessitates invasive procedures, such as the implantation of imaging windows, gradient-index (GRIN) lenses, or optical fibers.^8–10^

Bioluminescence is a naturally occurring photon-generation mechanism in which a luciferase enzyme uses molecular oxygen to oxidize a luciferin substrate, producing light.^11–13^ The amount of light generated through bioluminescence is relatively low and is generally considered safe for biological systems. Although bioluminescence imaging (BLI) has limited spatiotemporal resolution, it complements the shortcomings of fluorescence imaging.^14,15^ Unlike fluorescence imaging, bioluminescence imaging does not require external excitation, making it inherently compatible with photon-sensitive biological processes and optogenetic methods. Furthermore, bioluminescence creates a self-illuminating light source, making it particularly well-suited for minimally invasive imaging in deep tissues.

NanoLuc (NLuc) is an engineered luciferase derived from the functional subunit of *Oplophorus* luciferase (OLuc).^16^ It features a miniaturized protein size (∼19.2 kDa) and high brightness, making it an excellent choice for bioluminescence imaging. Over the past decade, NanoLuc and its derivatives have been effectively developed into numerous bioluminescent indicators.^17–21^ Given the pivotal role of Ca^2+^ in neuronal activity and the potential of bioluminescent imaging for minimally invasive monitoring of specific neuronal populations in animals, developing bioluminescent indicators for Ca^2+^ has been a highly sought-after topic in the field.^7,17–26^ Despite the progress, only a few bioluminescent Ca^2+^ indicators are suitable for in vivo animal imaging since it requires the superior transmission of long-wavelength red light in mammalian tissues. Our previously reported indicator, BRIC (bioluminescent red indicator for Ca^2+^), stands out due to its superior brightness, response magnitude, and red-shifted emission, making it one of the top choices for in vivo BLI (Table S1).^19^ However, despite the progress, the performance of bioluminescent Ca^2+^ indicators still lags significantly behind fluorescent Ca^2+^ sensors.

Herein, we report the engineering of an enhanced Ca^2+^ sensor, eBRIC, derived from the original BRIC through three rounds of directed evolution. Compared to BRIC, eBRIC exhibits a significantly improved Ca^2+^ response in vitro, as well as in cultured mammalian cell lines and primary neurons. To extend its application to live animal models, we used eBRIC to record Ca^2+^ dynamics in the somatosensory cortex and BLA regions in live mice, showcasing the ability of eBRIC for capturing the real-time activity of a neuronal population in a physiologically relevant context.

## Methods

### General Information

All animal studies were carried out per the Institutional Animal Care and Use Committees’ approvals at the University of Virginia (Protocol #4196) and Cornell (Protocol #2015-0029). BALB/cJ mice (#000651) and C57BL/6 J mice (#000664) were procured from the Jackson Laboratory and bred and maintained under standard conditions. DTZ and sDTZ luciferins were chemically synthesized according to the previously reported procedures.^27,28^

### Engineering and In Vitro Characterization of eBRIC

Random mutations were introduced into BRIC in a pBAD/HisB vector via error-prone PCRs,^29^ and the resultant libraries were screened as previously described^19^ except for the following modification. After preparing cell lysates, 5 μL of the supernatant was mixed with 185 μL of a buffer providing 65 nM free Ca^2+^ (30 mM MOPS, 100 mM KCl, 8.75 mM EGTA, 1.25 mM CaEGTA, pH 7.2) or another buffer providing 1.35 μM free Ca^2+^ (30 mM MOPS, 100 mM KCl, 5 mM EGTA, 5 mM CaEGTA, pH 7.2). Ten μL of DTZ solution (500 μM) was dispensed into each well using a reagent injector in a BMG Labtech CLARIOstar Plus microplate reader, resulting in a final DTZ concentration of 25 μM. The bioluminescence spectrum of each well was recorded from 450 to 700 nm with 10 nm intervals. Mutants exhibiting both high brightness and Ca^2+^ responsiveness were chosen for further analysis. Through three rounds of directed evolution, eBRIC was developed. The expression, purification, concentration determination, and in vitro characterization of the eBRIC protein were conducted following the established procedures used for BRIC.^19^

### Characterization of eBRIC in Mammalian Cell Lines

The gene for eBRIC was cloned into pcDNA3 to create pcDNA3-eBRIC, with pcDNA3-BRIC^19^ utilized for comparison. Next, 3 μg of plasmid DNA was used to transfect HeLa cells (ATCC, Cat. # CCL-2) in each 35 mm culture dish. After overnight incubation at 37 °C in a 5% CO_2_ incubator, cells were rinsed twice with DPBS (no Ca^2+^ and Mg^2+^), followed by a 20 min incubation in DPBS before imaging using an inverted Leica DMi8 microscope equipped with a Photometrics Prime 95B Scientific CMOS camera and controlled by Leica LAS X (Version 3.5.7) software. Bioluminescence was initiated by exchanging DPBS with fresh DPBS containing 100 μM DTZ. Imaging parameters included a 40× oil immersion objective lens (NA 1.2), no filter cube, 2 × 2 camera binning, 1 s exposure with 5 s intervals, camera sensor temperature set at −20 °C, and camera in 12-bit high sensitivity mode. Histamine, dissolved in DPBS, was introduced at a final concentration of 20 μM during the time-lapse imaging. Image processing and data analysis were conducted following established protocols.^19^ The experiments with HEK 293T cells (ATCC, Cat. # CRL-3216) were performed similarly, except that a luminescence imaging buffer (0.49 mM MgCl_2_, 2 mM CaCl_2_, 0.4 mM MgSO_4_, 0.44 mM KH_2_PO_4_, 5.3 mM KCl, 4.2 mM NaHCO_3_, 0.34 mM Na_2_HPO_4_, 138 mM NaCl, 10 mM HEPES pH 7.2, 15 mM d-glucose, and 0.1 mM sodium pyruvate) was used instead of DPBS. In addition, the images were acquired with 2 s exposure and 25 s intervals. Acetylcholine (Thermo Scientific, Cat. # AC159170050), dissolved in the luminescence imaging buffer, was introduced at a final concentration of 10 μM.

### Characterization of eBRIC in Primary Mouse Neurons

The production of adeno-associated viruses (AAVs), neuron preparation, transduction, and culture procedures were conducted following established protocols.^19^ Neurons expressing eBRIC and BRIC were assessed on the fifth day post-transduction with the corresponding AAVs. Prior to imaging, the growth medium was substituted with 1.6 mL of the luminescence imaging buffer supplemented with 100 μM DTZ. During time-lapse imaging, 0.42 mL of high K^+^ stimulation buffer (0.49 mM MgCl_2_, 2 mM CaCl_2_, 0.4 mM MgSO_4_, 0.44 mM KH_2_PO_4_, 143.2 mM KCl, 4.2 mM NaHCO_3_, 0.34 mM Na_2_HPO_4_, 10 mM HEPES pH 7.2, 15 mM d-glucose, and 0.1 mM sodium pyruvate) was applied to depolarize neurons. The imaging setup and data analysis were consistent with the previous section, except for an exposure time of 2 s with 3 s intervals.

### Surgery Preparation and Bioluminescence Recording of Somatosensory Cortex Ca^2+^ Dynamics in Mice

C57BL/6J mice (*n* = 6) were anesthetized with isoflurane and secured in a stereotaxic frame with a heating pad. A 4 mm craniotomy was made above the whisker barrel cortex (AP: −1 to −1.5 mm; ML: 3.5–4.0 mm), as described previously.^30^ AAV-hSyn-eBRIC (5 × 10^13^ GC/mL) was injected into four sites (250 nL each at 2 nL/s) using a glass micropipette. A PDMS cranial window was placed over the cortex and sealed with Vetbond (3M), followed by skull reinforcement with Metabond (Parkell) and attachment of a titanium headplate.^31^ Mice recovered for at least 3 weeks before imaging. For imaging, mice were anesthetized with isoflurane (4% induction, ∼0.5% maintenance). Laser speckle imaging confirmed functional activation in the whisker barrel cortex.^32^ Expression of eBRIC was verified using a custom-built two-photon microscope (920 nm excitation) under 4× and 20× (ZEISS W Plan-Apochromat lens, NA 1.0) objectives. For intravenous DTZ delivery, the tail was disinfected with 70% ethanol and warmed. A catheter (26G needle with polyurethane intravascular tubing (BTPU-027) prefilled with 10 U/mL heparinized saline) was inserted into a lateral tail vein, and 10 μL of saline was flushed to prevent clotting. The catheter was secured with medical tape, and the mouse was head-fixed onto a stereotaxic stage via the implanted headplate. Three needle electrodes were inserted into the whisker pad and connected to an Isolated Pulse Stimulator (Model 2100, A-M Systems); one served as ground, and the others targeted distinct whisker pad regions. After locating the area of viral expression via two-photon fluorescence, the excitation laser was blocked, and bioluminescent emission was captured using the microscope detectors with a 645/65 filter on photomultiplier tube (Hamamatsu, H10770B-50) with a home-built preamplifier (I to V gain 2 × 10^5^ Ohms; ∼10 MHz bandwidth). A 5 mL injection buffer for DTZ was made by dissolving 1.25 g (2-hydroxypropyl)-β-cyclodextrin (HP-β-CD) and 1 mL PEG-400 in ∼3 mL normal saline. DTZ was then dissolved in this buffer to a final concentration of 2.5 mM. DTZ was infused into mice at 25 μL/min via a syringe pump (KD Scientific). Electrical stimulation (5 Hz, 5 s, 2 mA and 4 mA) was delivered through the electrodes. Following imaging, electrodes and catheter were removed, the tail was disinfected, and the mouse was returned to its home cage.

### BLI of BLA Ca^2+^ Dynamics in Awake Mice

One μL of AAV-hSyn-eBRIC (5 × 10^13^ GC/mL) or an equivalent amount of AAV-hSyn-BREP was bilaterally injected into the BLA brain region (coordinates relative to Bregma: AP −3.4, ML ±1.25, DV −4.9)^19^ of 8-week-old BALB/cJ mice. BLI was conducted 3 weeks postviral administration. Before imaging, each awake mouse received a tail vein injection of 100 μL of sDTZ (25 mM) in normal saline. The mouse was then secured in a Narishige plastic mouse head holder (SRP-AM2) for imaging. BLI was performed using a UVP BioSpectrum dark box, a Computar Motorized ZOOM lens (M6Z1212MP3), and a Photometrics Evolve 16 EMCCD camera. The camera settings included an EM gain of 1000, 8 × 8 binning, an exposure time of 100 ms without intervals, and a sensor temperature of −70 °C. The mice were positioned 27 cm from the lens. Each experimental session consisted of a 100 s acclimation period for the animals, followed by 13 footshock trials. Each trial involved a 0.8-mA electric footshock lasting 1 s, with 40 s intervals between shocks, administered using an A-M Systems 2100 isolated pulse stimulator. Image processing and data analysis were performed according to an established procedure.^19^

## Results

### Engineering and Characterization of eBRIC In Vitro

BRIC is a bioluminescent Ca^2+^ indicator generated by inserting the Ca^2+^-sensory calmodulin (CaM) and M13 moieties into a NanoLuc-derived teLuc luciferase, linked to the red fluorescent protein mScarlet-I.^19^ The indicator has more than half of its emission above 600 nm due to bioluminescent resonance energy transfer (BRET) from the Ca^2+^-regulated luciferase to the red fluorescent protein. In vitro, it showed an excellent 5.5-fold ΔBL/BL_0_ response. Although BRIC has been established as a benchmark for BLI of Ca^2+^, its cellular responses require further improvement.

Built upon the success of BRIC, we aimed to further improve its responsiveness to physiological Ca^2+^ fluctuations. To achieve this, we conducted random mutagenesis on BRIC and screened the resulting libraries using 65 nM and 1.35 μM of free Ca^2+^, which differs from previous screening approaches that used 0 and 39 μM of free Ca^2+^.^17,19^ This new range of Ca^2+^ concentrations was chosen because it better matches the cytosolic Ca^2+^ concentrations of mammalian cells at rest and excited states. Through three rounds of directed evolution, we successfully generated a drastically enhanced BRIC (eBRIC) variant with an 18.2-fold Ca^2+^-included bioluminescence increase in cell lysates (Figure 1A). We also measured the brightness of BRIC and eBRIC in both the Ca^2+^-free (off) state and the Ca^2+^-bound (on) states using bacterial lysates. Compared to BRIC, eBRIC displayed reduced brightness in the Ca^2+^-free state but enhanced brightness in the Ca^2+^-bound state (Figure S2).

**Figure 1:**
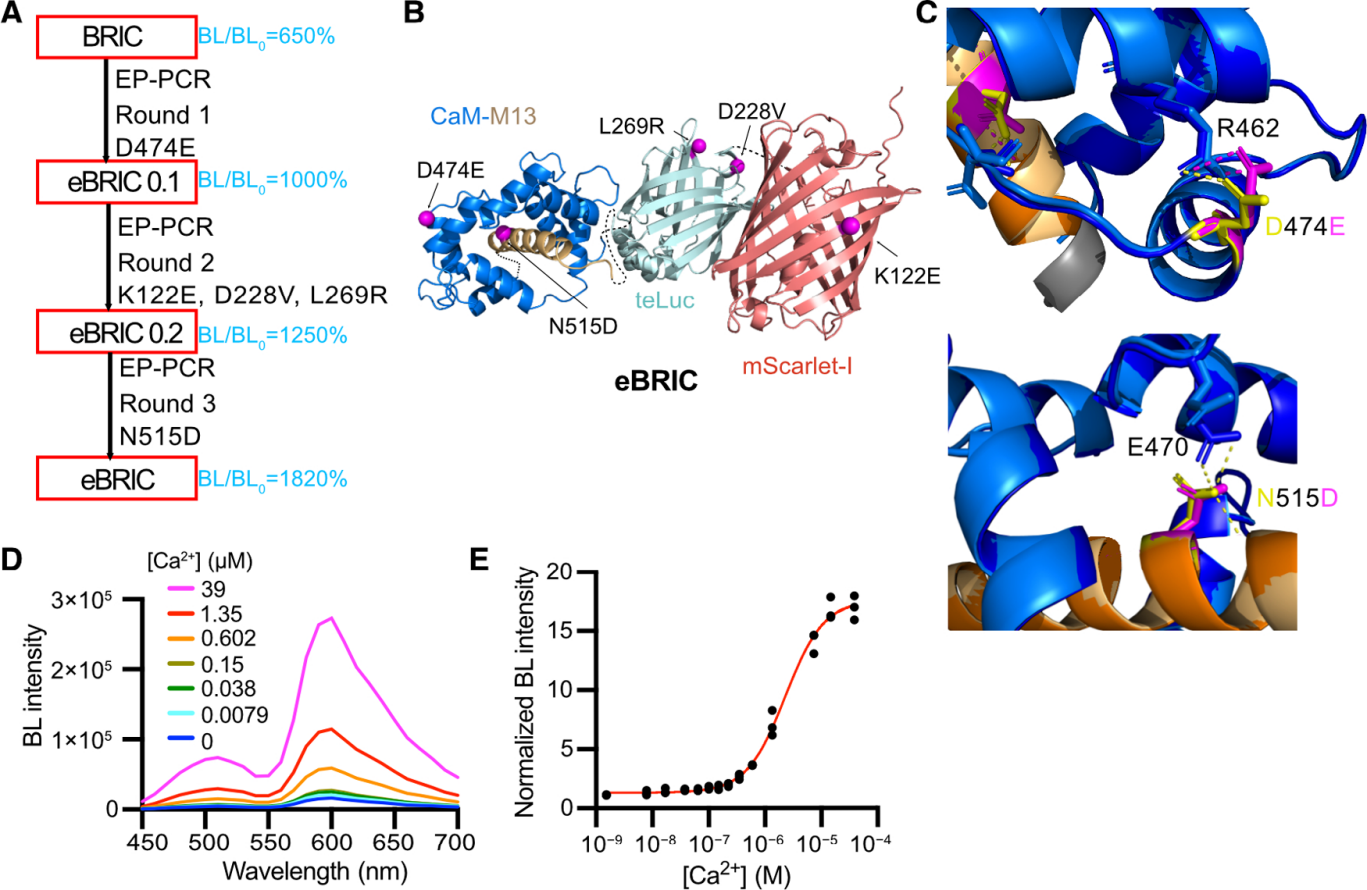
Engineering and in vitro characterization of eBRIC. (A) Flowchart showing the engineering of eBRIC from BRIC. The ratios of bioluminescence in the presence of 39 μM Ca^2+^ to the absence of Ca^2+^ are also included, as determined from bacterial lysate-based assays. (B) Domain arrangement of eBRIC with five mutations from BRIC highlighted as magenta balls. The CaM (calmodulin), M13, teLuc, and mScarlet-I domains are colored in marine blue, golden yellow, pale cyan, and salmon pink, respectively. (C) Highlight of mutations on CaM and M13. ColabFold v1.5.5 was used to generate model structures for BRIC and eBRIC. The CaM portions in BRIC and eBRIC are colored blue and cyan, respectively, while the M13 portions in BRIC and eBRIC are colored yellow and light orange, respectively. D474 and N515 in BRIC are shown in orange, while E474 and D515 in eBRIC are shown in magenta. (D) Bioluminescence spectra of eBRIC in the presence of DTZ and varying concentrations of free Ca^2+^. (E) Ca^2+^-dependency of eBRIC bioluminescence at 600 nm (*n* = 3 technical replicates). A four-parameter Hill equation was used to fit the data to determine the dissociation constant (*K*_d_ = 2.3 μM and Hill coefficient = 1.3).

During this process, five mutations were identified: two in teLuc (L269R and D228 V), one in mScarlet-I (K122E), and two in the CaM-M13 region (D474E and N155D) (Figure 1B and Supporting Information Figure S1). Notably, the two mutations in the CaM-M13 region are primarily responsible for the observed response enhancement (Figure 1A). Although it is challenging to precisely understand the underlying mechanisms, based on our ColabFold^33^-generated models, residue 474 is involved in a hydrogen bond with Arg462, bridging two α-helices close to two Ca^2+^ binding sites. Residue 155 forms a hydrogen bond with E470, facilitating interactions between M13 and CaM (Figure 1C). Presumably, these mutations help maximize the structural and activity differences between the Ca^2+^-free and Ca^2+^-bound states of the sensor.

We further performed spectroscopic characterization using purified proteins. From BRIC to eBRIC, the response between 65 nM and 1.35 μM of Ca^2+^ increased from 1.7-fold to 4.6-fold, while the overall Ca^2+^-induced change between 0 and 39 μM of free Ca^2+^ increased from 6.5-fold to ∼17-fold (Figure 1D). The apparent dissociation constant (*K*_d_) also increased from 133 nM to 2.3 μM (Figure 1E). Collectively, these results indicate that eBRIC is a high-quality Ca^2+^ sensor with the potential for sensitively detecting Ca^2+^ changes in the physiologically relevant concentration range (Table S1).

### Characterization of eBRIC in Cultured Mammalian Cells and Primary Neurons

Next, we tested eBRIC for imaging Ca^2+^ dynamics in HeLa cells. 100 μM DTZ was added to initiate bioluminescence. In eBRIC-cells, the stimulation with 20 μM histamine resulted in robust bioluminescence oscillations, which indicates Ca^2+^ oscillations (Figure 2A,B and Movie S1). Comparing this to BRIC at identical conditions, the magnitude of the change (ΔBL/BL_0_) was nearly 5-fold higher. To test whether eBRIC is functional in other cell types, we expressed eBRIC in HEK 293T cells and stimulated them with 10 μM of acetylcholine. We observed a robust 3.5-fold (ΔBL/BL_0_) bioluminescence increase, indicating a successful detection of acetylcholine-induced Ca^2+^ increases in the cytol (Figure 2C). Finally, we compared eBRIC and BRIC in primary mouse neurons. high K^+^ depolarization induced a 7.3-fold increase (ΔBL/BL_0_) in eBRIC bioluminescence, resulting in a 5.6-fold increase in responsiveness from BRIC (Figure 3 and Movie S2).

**Figure 2:**
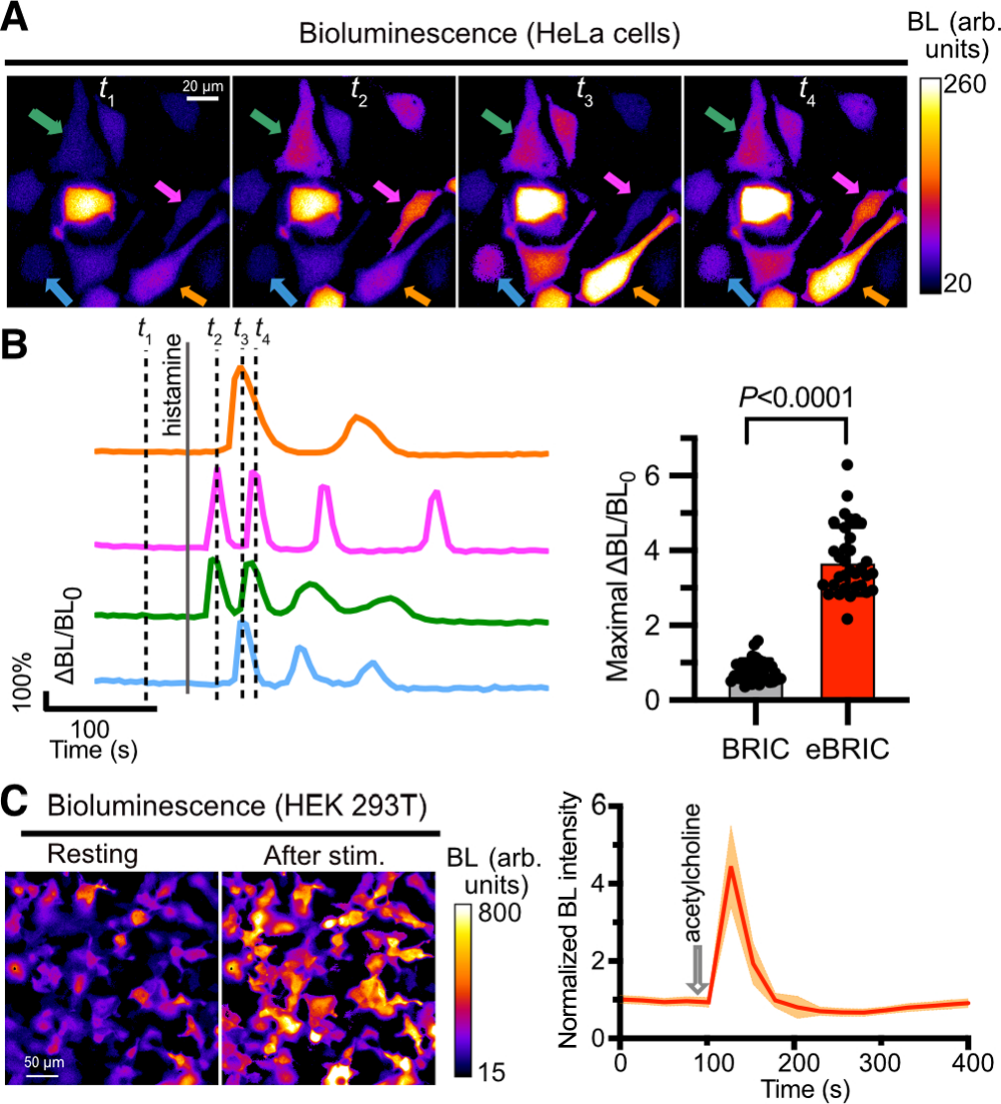
Imaging Ca^2+^ dynamics in cultured mammalian cell lines using eBRIC. (A) Representative pseudocolored bioluminescence images of histamine-induced Ca^2+^ dynamics in HeLa cells. Scale bar, 20 μm. (B) Left: Intensity traces for individual cells. The color of the traces corresponds to the colors of the arrows used to highlight individual cells in the left images. The baselines of the traces were corrected for monoexponential decay resulting from substrate consumption. Dashed lines indicate the four time points corresponding to the images shown in panel A, and the timing of histamine addition is marked by the gray line. Right: Parallel comparison between eBRIC and BRIC in terms of the maximal changes (ΔBL/BL_0_) of individual cells induced with histamine. Data are expressed as mean ± s.d. (*n* = 33 cells for eBRIC and 34 cells for BRIC), with the *P* value derived from unpaired two-tailed *t* tests. (C) Left: Representative pseudocolored bioluminescence images of eBRIC-expressing HEK 293T cells before and after acetylcholine stimulation. Right: Intensity traces for individual cells. Data are expressed as mean ± s.d. (*n* = 20 cells).

**Figure 3:**
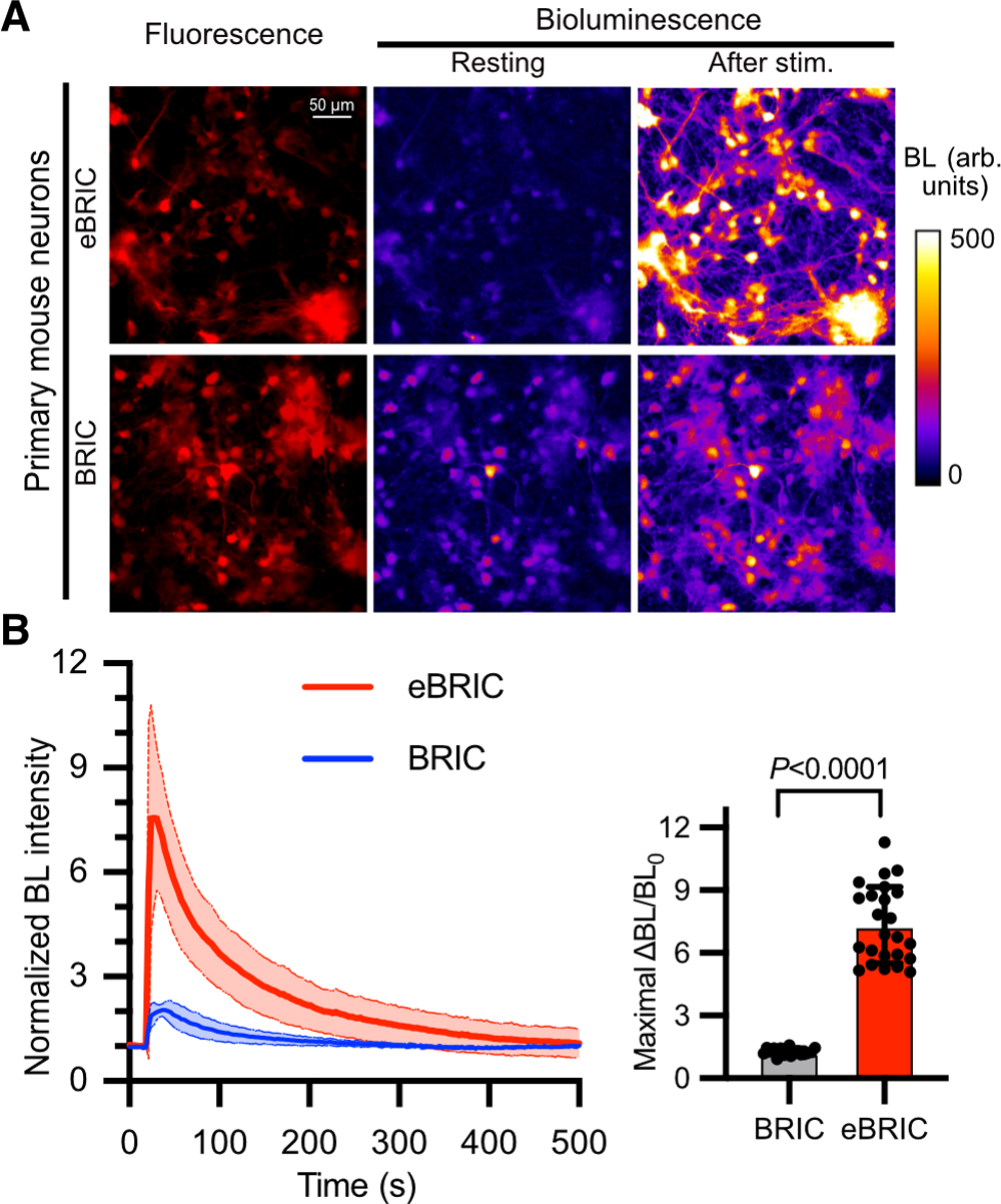
Comparison of BRIC and eBRIC for imaging Ca^2+^ influx during the depolarization of cultured primary mouse neurons. (A) Representative fluorescence and bioluminescence images of primary mouse neurons expressing eBRIC or BRIC in response to high K^+^ (30 mM) depolarization. Scale bar, 50 μm. (B) Left: Quantification of the responses of eBRIC- or BRIC-expressing neurons. Right: Comparison of the maximal changes (ΔBL/BL_0_) of individual cells. Data are expressed as mean ± s.d. (*n* = 24 cells for eBRIC and 17 for BRIC), with the *P* value determined through unpaired two-tailed *t* tests.

The brightness of bioluminescent indicators is a critical parameter for effective imaging. We compared the brightness of eBRIC to BRIC in both transfected HeLa cells and AAV-transduced primary mouse neurons (Figure S2). In HeLa cells, we measured brightness in both the Ca^2+^-free (off) state and the Ca^2+^-bound (on) state following histamine stimulation. eBRIC exhibited reduced brightness in the Ca^2+^-free state but demonstrated approximately twice the brightness of BRIC after stimulation. A similar trend was observed in primary neurons. To further evaluate the performance of eBRIC in mammalian systems, we measured its bioluminescence emission spectrum in HEK 293T cells (Figure S3). The spectral profile closely resembled that of eBRIC in lysates and as a purified protein, suggesting that expression in mammalian cells does not alter the BRET efficiency.

Taken together, eBRIC has proven to be a highly effective bioluminescent indicator for detecting physiologically relevant Ca^2+^ changes in living cells.

### In Vivo BLI of Ca^2+^ Dynamics in Live Mice

To validate the functionality of eBRIC for recording Ca^2+^ dynamics in the mouse brain, we performed microinjections of AAV-hSyn-eBRIC into craniotomies targeting the whisker barrel region of the somatosensory cortex. A few weeks postinjection, two-photon microscopy was used to confirm sensor expression, and the same setup was used to record bioluminescence emission during sensory stimulation with the excitation laser blocked (Figure 4A). Two-photon excited fluorescence revealed robust eBRIC expression in neuronal somata and processes. Neurons appeared healthy and morphologically intact even three months after surgery, indicating minimal toxicity associated with viral expression (Figure 4B). Laser speckle contrast imaging was used to confirm that the viral expression was in a region that responded to electrical stimulation of the whisker pad (Figure 4C).^32^ Bioluminescence signals were recorded through the objective of the two-photon microscope positioned over the center of the eBRIC-expressing region identified by fluorescence. A 2.5 mM DTZ solution was infused via a tail vein catheter while electrical stimulation was applied through electrodes at two distinct locations of the whisker pad using 2-mA and 4-mA pulses (5 Hz, 5 s). Infusion at 25 μL/min generated a steady level of baseline bioluminescence and clear increases in bioluminescence were observed with stimulation (4 and 2 mA) at both electrodes on the whisker pad (left and right panels, orange orange and blue traces in Figure 4D), with the 4 mA condition producing a sufficiently strong response to detect individual stimulation events. Notably, subtle differences emerged between the responses evoked by stimulation with the two electrodes, suggesting that some of the virally labeled neurons may be selectively responsive to specific stimulus locations. Stimulation of region 1 resulted in a delayed peak response, while region 2 elicited an earlier rise in signal following stimulation onset.

**Figure 4:**
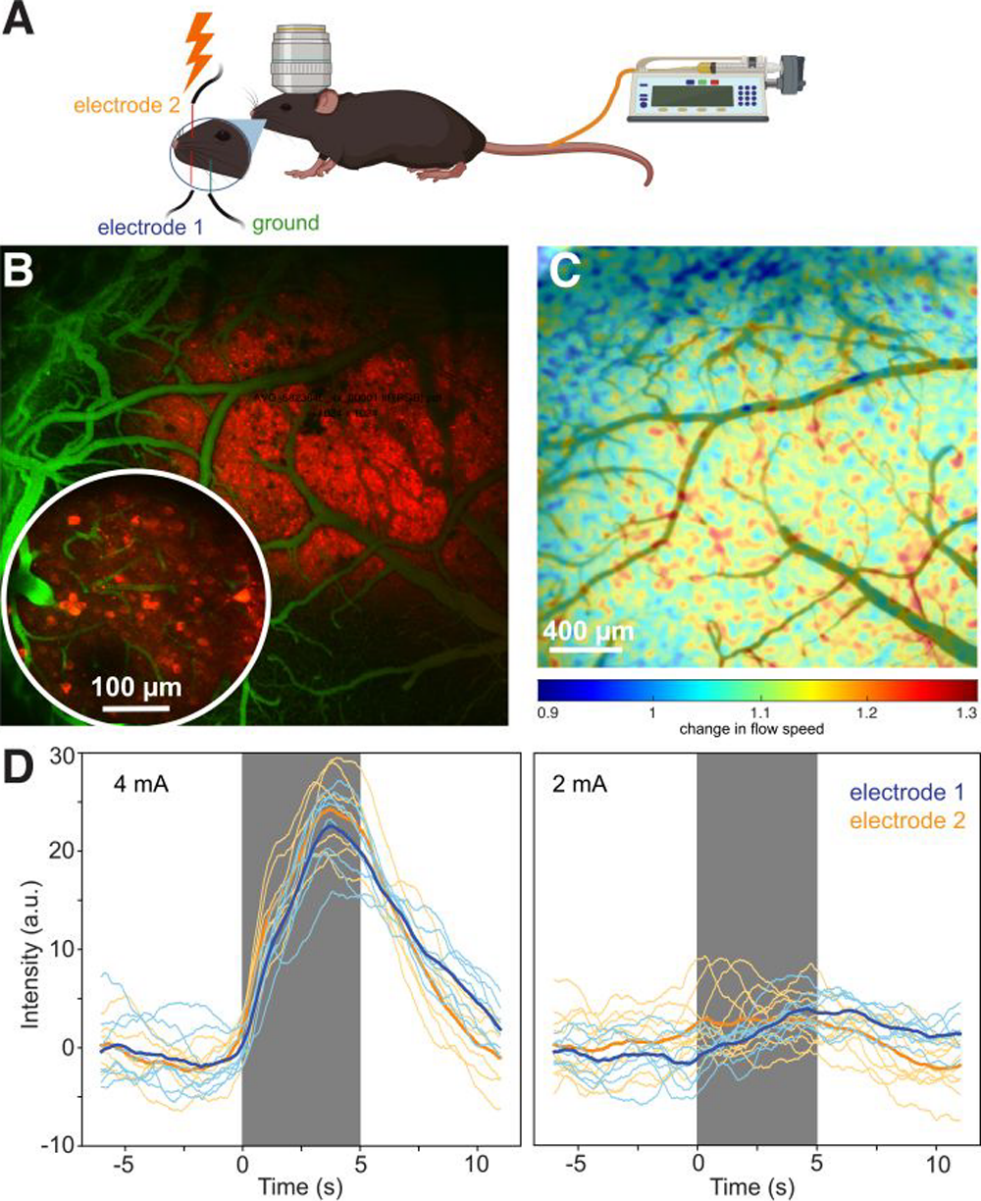
In vivo recording of stimulus-evoked neural activity using eBRIC bioluminescence in mouse somatosensory cortex. (A) Schematic of the experimental setup. Bioluminescence was recorded through a cranial window using the objective and detection optics of a two-photon microscope. Inset illustrates whisker pad stimulation with placement of stimulation and ground electrodes. (B) Two-photon fluorescence image of eBRIC (red, with 4× objective), showing the virally transduced region in the whisker barrel cortex. Blood vessels were labeled with FITC-dextran (green). The circular inset highlights representative labeled neurons (with 20× objective) in an average projection spanning 30 μm in the *z*-axis. (C) Laser speckle contrast imaging of the same region in panel B, showing blood flow changes in response to stimulation. Vessels are overlaid in gray. (D) Evoked bioluminescence signals during whisker pad stimulation (gray rectangle) in a representative mouse, recorded at stimulation currents of 4 mA (left) and 2 mA (right). Light traces represent individual trials, while bold traces indicate averaged responses. Blue and orange colors denote two different electrode locations. Traces are smoothed using a 1-s moving average.

To further demonstrate the use of eBRIC for in vivo imaging of the activity of neuronal ensembles in awake animals, we used an AAV carrying the eBRIC gene to infect neurons in the basolateral amygdala (BLA) region of the brains of live mice. We paired eBRIC with our recently developed water-soluble sDTZ luciferin^28^ for bioluminescence whole-animal imaging. The BLA is known to play a crucial role in generating fear-associated behaviors,^34^ and aversive stimuli such as footshock are expected to activate neurons in this region. Prior to imaging, each mouse received a tail vein injection of 100 μL sDTZ (25 mM) dissolved in normal saline. During imaging, mice were subjected to repeated 1-s footshock stimulations at 0.8 mA, delivered at 40-s intervals. As expected, we observed a significant and consistent increase in bioluminescence in the eBRIC group in response to footshocks (Figure 5 and Movie S3). As a negative control, we generated mice expressing BREP,^19^ a Ca^2+^-insensitive bioluminescent protein, and observed minimal to no changes in bioluminescence under electric footshock stimulation. The combination of eBRIC and sDTZ allowed digitalization at a frequency of 10 Hz, which has not been achieved in previous studies. Compared to prior experiments with BRIC under the same stimulation condition,^19^ the response magnitude of eBRIC has nearly doubled. These findings confirm that eBRIC serves as a powerful tool for tracking the activity of neuronal populations with improved temporal resolution and sensitivity.

**Figure 5:**
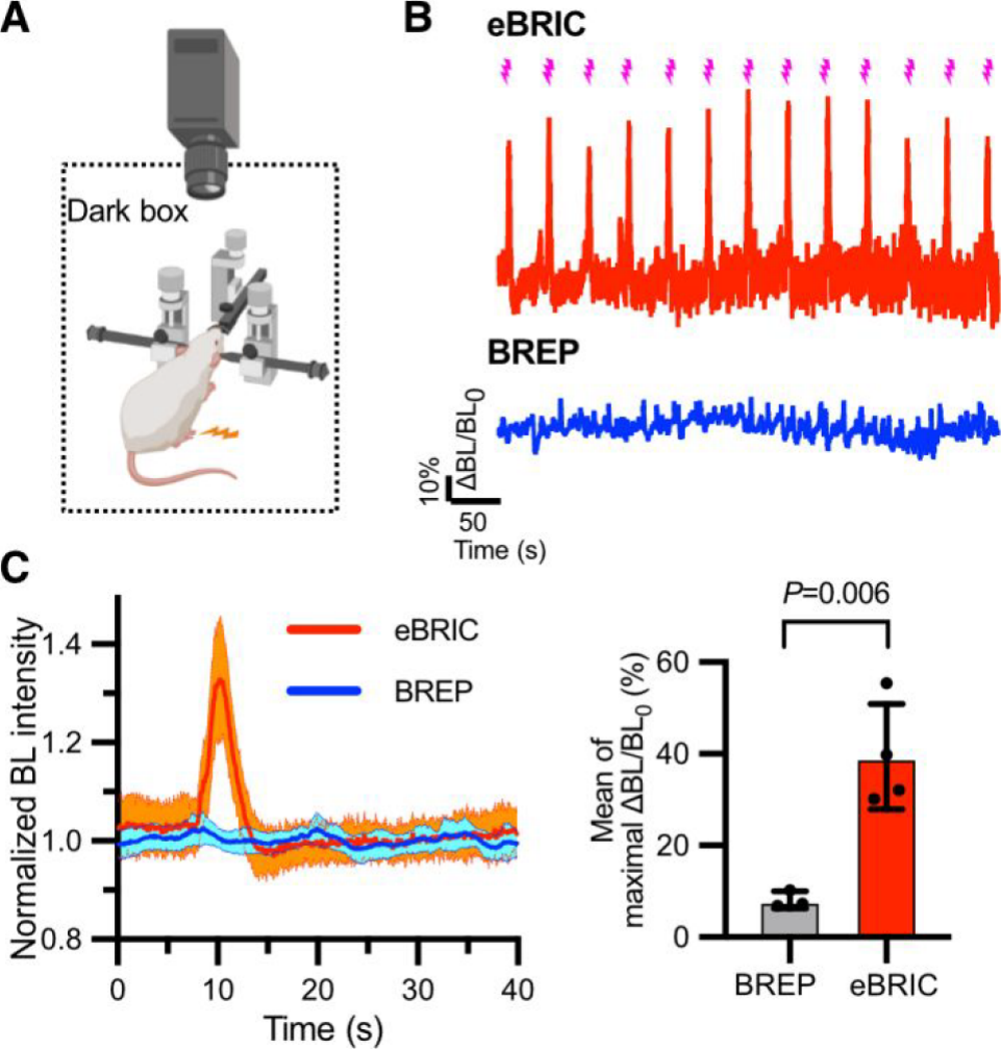
Imaging Ca^2+^ dynamics in head-fixed awake mice. (A) Scheme of BLI of head-fixed awake mice. eBRIC expression in the basolateral amygdala (BLA) region was achieved using AAV vectors. Prior to imaging, sDTZ was administered to mice via the tail vein. Footshocks were then employed as a means of stimulation. Images were acquired with a 100 ms exposure time without any intervals. (B) Representative bioluminescence intensity traces for mice expressing either eBRIC or BREP (negative control) following 13 consecutive footshock stimulation trials. (C) Left: Quantification of intensity changes of eBRIC- and BREP-expressing mice in response to footshock. Data is presented as mean ± s.d. and based on images of 4 mice for eBRIC and 3 mice for BREP, each consisting of 13 trials. Right: Comparison of the eBRIC and BREP groups by averaging the responses of individual mice over 13 trials. Data are expressed as mean ± s.d. (*n* = 4 mice for eBRIC and 3 mice for BREP), with the *P* value derived from unpaired two-tailed *t*-tests. This figure is created with BioRender.com.

## Discussion

Bioluminescent Ca^2+^ indicators hold great promise as tools for functional imaging of bioactivities. In contrast to fluorescent indicators, bioluminescent indicators provide the advantage of being less invasive. However, a key limitation of existing bioluminescent Ca^2+^ indicators is their relatively low responsiveness. Here we developed an enhanced bioluminescent Ca^2+^ indicator (eBRIC) based on BRIC by using a physiological Ca^2+^ concentration range during library screening. We successfully optimized eBRIC to exhibit significantly improved performance. In vitro characterization and experiments in mammalian cells and animals revealed that eBRIC demonstrates increased sensitivity to physiological Ca^2+^ levels.

Although the *K*_d_ of eBRIC has increased to 2.3 μM, it exhibits a higher fold of bioluminescence changes within the range of 65 nM to 1.35 μM Ca^2+^. Subsequently, eBRIC demonstrated favorable performance in both cellular and animal models. Compared to Orange CaMBIs,^17^ we gained 7.4- to 18-fold increase in Ca^2+^ responsiveness using the new eBRIC Ca^2+^ sensor in physiologically stimulated live cells (Table S1). Compared to our previously reported BRIC sensor,^19^ eBRIC is 4.5- to 5.6-fold more responsive (Table S1). As BRIC has been set as a benchmark for bioluminescent Ca^2+^ indicators,^19^ eBRIC signifies a substantial advancement beyond BRIC. Indeed, the combination of eBRIC with our recently developed luciferin substrate, sDTZ, expands the capability of functional neuronal imaging. The successful demonstration of minimally invasive, video-rate imaging of Ca^2+^ activity in a defined brain region in awake mice represents a significant milestone, underscoring the potential of eBRIC as a powerful tool for investigating dynamic processes in living organisms.

While eBRIC demonstrates clear performance gains over its predecessor, the substantial response enhancement observed in cultured cells translated to a more modest improvement in the footshock-induced BLA activation paradigm in animals. The underlying reasons for this discrepancy are not fully understood, but may include factors such as limitations in substrate availability, measurement noise, hemodynamic and temperature effects, and variability in sensor expression or folding. It is worth noting that similar reductions in performance have been frequently observed with other fluorescent protein–based biosensors when transitioning from in vitro to in vivo settings.^35^ Thus, to overcome these limitations, more frequent iterative testing between in vitro and in vivo systems—or approaches that enable direct engineering and optimization of indicators within animal models—may be necessary. Additionally, while eBRIC produces greater bioluminescence than BRIC in the Ca^2+^-bound state, its reduced brightness in the Ca^2+^-free state may limit the signal-to-noise ratio under basal conditions. When paired with sDTZ, which can be administered at a concentration of 25 mM, eBRIC enabled time-lapse imaging at 10 Hz. However, in vivo applications remain limited by the inherent trade-off between absolute signal intensity and achievable spatial-temporal resolution—particularly in deep tissue, where photon attenuation significantly reduces sensitivity. Although the temporal resolution represents an improvement, it may still fall short for accurately capturing rapid, transient Ca^2+^ dynamics.

We also recorded bioluminescence from live-animal brain activity using a microscope setup with the excitation light blocked. These results demonstrate that eBRIC can reliably report neural activity evoked by somatosensory stimulation in the cortex of a mouse implanted with a chronic cranial window and systemically administered substrate. Notably, we observed clear neural responses to single whisker pad shocks in individual trials, underscoring the high signal-to-noise ratio of eBRIC in vivo. Although a two-photon microscope was used to confirm sensor expression, the actual bioluminescence recordings do not require such specialized instrumentation. Since fluorescence microscopes are widely accessible, researchers can readily adapt this recording format for their own studies. Additionally, we used a syringe pump to infuse the DTZ substrate continuously, which provided more sustained bioluminescence signals compared to traditional bolus injections—a strategy that may prove useful for extended imaging sessions.

In parallel with our work, a recent preprint reported a promising bioluminescent Ca^2+^ indicator called CaBLAM, which is based on a newly developed SSLuc luciferase.^36^ In validation experiments, CaBLAM demonstrated robust and impressive responses. Unlike eBRIC, CaBLAM emits in the blue range and does not incorporate a red fluorescent protein for BRET-based red shifting. As a result, imaging was performed using a wide-field microscope equipped with an EMCCD camera, under no external illumination, targeting superficial cortical regions accessible through cranial windows. Nonetheless, eBRIC should also be compatible with such imaging setups. Future studies directly comparing eBRIC and CaBLAM would be valuable for benchmarking their relative strengths and limitations under various experimental conditions and for guiding the development of next-generation bioluminescent indicators.

In conclusion, the development of eBRIC signifies a leap forward in bioluminescent imaging technology. Its high sensitivity, enhanced response magnitude, and compatibility with less invasive imaging techniques make it a powerful tool for investigating dynamic processes in living organisms. We anticipate that eBRIC will become an invaluable asset for researchers, enabling new studies on biological processes, neuronal dynamics, and disease mechanisms.

## Supplementary Material









## Notes

Sequence alignment and additional spectral characterization (PDF)
Movie S1, bioluminescence imaging of histamine-induced Ca^2+^ dynamics in eBRIC-expressing HeLa cells (MP4)
Movie S2, bioluminescence imaging of primary mouse neurons expressing eBRIC in response to high K^+^ depolarization (MP4)
Movie S3, bioluminescence imaging of a head-fixed awake mouse with eBRIC expression in the BLA region following consecutive footshock stimuli (MP4)

## Abbreviations

AAV: adeno-associated virus
Arb. units: arbitrary units
BL: bioluminescence
BLI: bioluminescence imaging
BLA: basolateral amygdala
eBRIC: enhanced bioluminescence red indicator for Ca^2+^
*K*_d_: apparent dissociation constant
