# Supplementary material for: An Enhanced Red Bioluminescent Indicator for Responsive Detection of Physiological Calcium Dynamics in Cells and Mice
Source: ACS Sens. 2025 Jul 29;10(8):5826–33. doi: 10.1021/acssensors.5c01093 (PMC12351538; doi:10.1021/acssensors.5c01093)
Supplement: Supplementary file 1 [file se5c01093_si_001.pdf]

Supporting Information

An Enhanced Red Bioluminescent Indicator for Responsive Detection of Physiological Calcium Dynamics in Cells and Mice

Xiaodong Tian,[1,2] Yiyu Zhang,[1,2] Haoyang Du,[3] Wenyuan Huang,[1,2] Laurie Anne Bizimana,[3] Nozomi Nishimura,[3*] and Hui-wang Ai[1,2,4*]

[1] Department of Molecular Physiology and Biological Physics, University of Virginia School of Medicine, Charlottesville, Virginia 22908, USA.

[2] Center for Membrane and Cell Physiology, University of Virginia School of Medicine, Charlottesville, Virginia 22908, USA.

[3] Meinig School Biomedical Engineering, Cornell University, Ithaca, New York 14853, USA

[4] The UVA Comprehensive Cancer Center, University of Virginia, Charlottesville, Virginia 22908, USA.

*E-mail: huiwang.ai@virginia.edu (H.A.) and nn62@cornell.edu (N.N.)

```
        1                                      40
eBRIC   MVSKGEAVIKEFMRFKVHMEGSMNGHEFEIEGEGRPYE
BRIC    MVSKGEAVIKEFMRFKVHMEGSMNGHEFEIEGEGRPYE

        41                                     80
eBRIC   GTQTAKLKVTKGGPLPFSWDILSPQFMYGSRAFIKHPADI
BRIC    GTQTAKLKVTKGGPLPFSWDILSPQFMYGSRAFIKHPADI

        81                                     120
eBRIC   PDYYKQSFPEGFKWERVMNFEDGGAVTVTQDTSLEDGTLI
BRIC    PDYYKQSFPEGFKWERVMNFEDGGAVTVTQDTSLEDGTLI

        121                                    160
eBRIC   YEVKLRGTNFPPDGPVMQKKTMGWEASTERLYPEDGVLKG
BRIC    YKVKLRGTNFPPDGPVMQKKTMGWEASTERLYPEDGVLKG

        161                                    200
eBRIC   DIKMALRLKDGGRYLADFKTIYKAKKPVQMPGAYNVDRKL
BRIC    DIKMALRLKDGGRYLADFKTIYKAKKPVQMPGAYNVDRKL

        201                                    240
eBRIC   DITSHNEDYTVVEQYERSEGRHLDTLEVFVGDWRQTAGYN
BRIC    DITSHNEDYTVVEQYERSEGRHLDTLEDFVGDWRQTAGYN

        241                                    280
eBRIC   LSQILEQGGVSSLFQNLGVSVTPIQRIVRSGENGLKIDIH
BRIC    LSQILEQGGVSSLFQNLGVSVTPIQRIVLSGENGLKIDIH

        281                                    320
eBRIC   VIIPYEGLSGDQMGQIEKIFKVVYPVDNHHFKVILHYGTL
BRIC    VIIPYEGLSGDQMGQIEKIFKVVYPVDNHHFKVILHYGTL

        321                                    360
eBRIC   VIDGVTPNLIDYFGRPYEGIAVFDGKKITVTGTLIMHDQL
BRIC    VIDGVTPNLIDYFGRPYEGIAVFDGKKITVTGTLIMHDQL

        361                                    400
eBRIC   TEEQIAEFKEAFSLFDKDGDGTITTKELGTVMRSLGQNPT
BRIC    TEEQIAEFKEAFSLFDKDGDGTITTKELGTVMRSLGQNPT

        401                                    440
eBRIC   EAELQDMINEVDADGNGTIYFPEFLTMMARKMKDTDSEEE
BRIC    EAELQDMINEVDADGNGTIYFPEFLTMMARKMKDTDSEEE

        441                                    480
eBRIC   IREAFRVFDKDGNGYISAAQLRHVMTNLGEKLTEEEVDEM
BRIC    IREAFRVFDKDGNGYISAAQLRHVMTNLGEKLTDEEVDEM

        481                                    520
eBRIC   IREADIDGDGQVNYEEFVQMMTAKGGESKRRWKKDFIAVS
BRIC    IREADIDGDGQVNYEEFVQMMTAKGGESKRRWKKNFIAVS

        521                                    560
eBRIC   AANRFKKISSSGALELWKGNKIIDERLINPDGSLLFRVTI
BRIC    AANRFKKISSSGALELWKGNKIIDERLINPDGSLLFRVTI

        561          574
eBRIC   NGVTGWRLHERILA -
BRIC    NGVTGWRLHERILA -
```

Fig. S1. Sequence alignment of eBRIC and BRIC. Sequences originating from mScarlet-I, teLuc, calmodulin (CaM), and M13 are represented in red, cyan, blue, and orange, respectively. Linker residues are shown in gray, while mutations acquired during directed evolution are shaded in light orange.

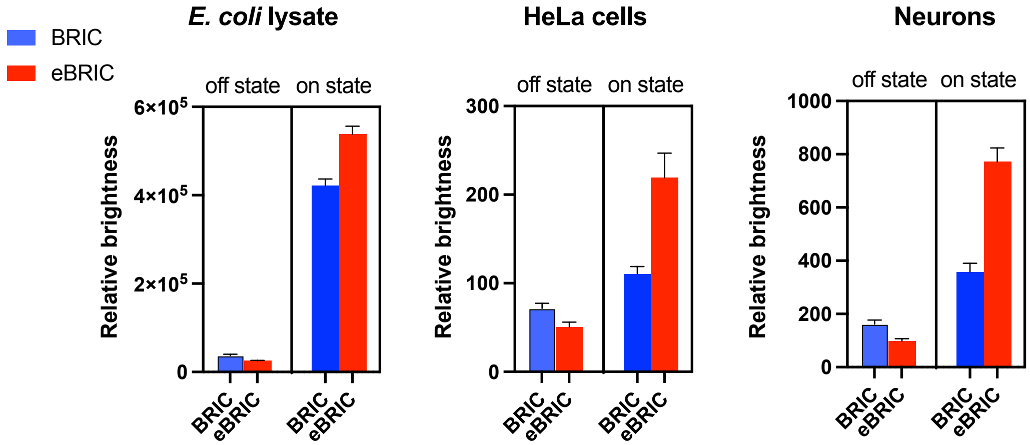

Fig. S2. Bioluminescence brightness comparisons between BRIC and eBRIC in *E. coli* lysate (off state: +EGTA; on state: 39 μM free Ca^{2+}), transiently transfected HeLa cells (off state: before stimulation; on state: after histamine stimulation), and AAV-transduced primary mouse neurons (off state: before stimulation; on state: after KCl-induced depolarization). Data are presented as mean ± s.e.m.

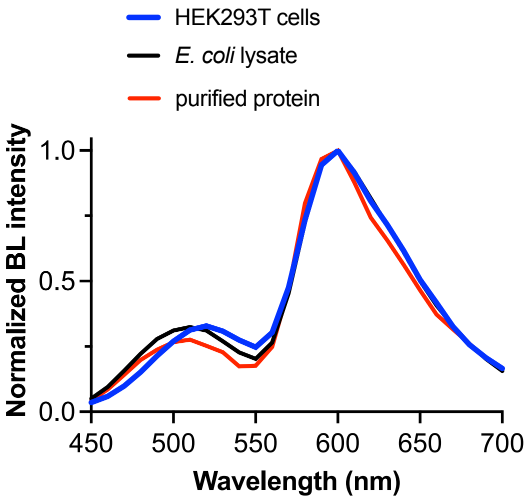

Fig. S3. Bioluminescence emission profiles of eBRIC under three conditions: as a purified recombinant protein (red trace), in *E. coli* lysate (black trace), and following transient expression in HEK 293T cells (blue trace).

Table S1. Characteristics of NanoLuc-derived bioluminescent Ca^{2+} indicators with notable >600 nm emission suitable for animal imaging.

	**Peak emission (nm)**	***K***	**Emission fraction >600 nm**	**$\Delta BL/BL_0$ *in vitro***	**$\Delta BL/BL_0$ in cells**	**Reference**
**Orange CaMBIs**	586	110-300	0.33	6	0.5 (HeLa†)<br>0.4 (neuron§)	1
**BRIC**	595	133	0.54	5.5	0.8 (HeLa†)<br>1.3 (neuron§)	2
**eBRIC**	595	2300	0.54	16	3.7 (HeLa†)<br>7.3(neuron§)	This work

†Comparison is made for maximal changes induced with 20 μM histamine.

§Comparison is made for maximal changes induced with 30 mM KCl.

References:

1. Oh, Y.; Park, Y.; Cho, J. H.; Wu, H.; Paulk, N. K.; Liu, L. X.; Kim, N.; Kay, M. A.; Wu, J. C.; Lin, M. Z., An orange calcium-modulated bioluminescent indicator for non-invasive activity imaging. *Nat. Chem. Biol.* **2019,** *15* (5), 433-436.
2. Tian, X.; Zhang, Y.; Li, X.; Xiong, Y.; Wu, T.; Ai, H.-w., A luciferase prosubstrate and a red bioluminescent calcium indicator for imaging neuronal activity in mice. *Nat Commun* **2022,** *13*, 3967.

Supplementary Movies

Movie S1 (separate file). Bioluminescence imaging of histamine-induced Ca^{2+} dynamics in eBRIC-expressing HeLa cells.

Movie S2 (separate file). Bioluminescence imaging of primary mouse neurons expressing eBRIC in response to high K^+ depolarization.

Movie S3 (separate file). Bioluminescence imaging of a head-fixed awake mouse with eBRIC expression in the basolateral amygdala (BLA) region following 13 consecutive footshock stimuli.
